# Behavioral Economic Assessment of Alcohol and Cigarette Demand in Smokers With Alcohol Use Disorder

**DOI:** 10.3389/fpsyt.2021.674607

**Published:** 2021-06-23

**Authors:** Yong Cui, Paulina Linares Abrego, Jin Ho Yoon, Maher Karam-Hage, Paul M. Cinciripini, Nassima Ait-Daoud Tiouririne, Robert M. Anthenelli, Jason D. Robinson

**Affiliations:** ^1^Department of Behavioral Science, The University of Texas MD Anderson Cancer Center, Houston, TX, United States; ^2^Department of Psychology, University of Houston, Houston, TX, United States; ^3^Department of Psychiatry and Behavioral Sciences, The University of Texas Health Science Center at Houston, Houston, TX, United States; ^4^Center for Addiction Research and Education, University of Virginia Health System, Charlottesville, VA, United States; ^5^Department of Psychiatry, University of California, San Diego, San Diego, CA, United States

**Keywords:** purchase task, alcohol demand, cigarette demand, latent structure, smokers with alcohol use disorder, alcohol and tobacco co-dependence

## Abstract

**Background and Objectives:** Behavioral economic purchase tasks are widely used to assess drug demand in substance use disorder research. Comorbid alcohol use is common among cigarette smokers and associated with greater difficulty in quitting smoking. However, demand for alcohol and cigarettes in this population has not been fully characterized. The present study addressed this gap by examining alcohol and cigarette demand among treatment-seeking smokers with alcohol use disorder (AUD).

**Methods:** Alcohol and cigarette demand was assessed among 99 smokers with AUD. We conducted Principal Component Analysis (PCA) and correlational analyses on the demand indices.

**Results:** Participants showed higher demand for alcohol than for cigarettes, as evidenced lower elasticity (resistance to increasing price) and higher O_max_ (maximum response output for drug). PCA revealed a two-factor structure (Persistence and Amplitude) for both alcohol and cigarette demand indices. Cigarette-related demand indices were positively correlated with nicotine dependence, but alcohol-related demand indices were not associated with alcohol dependence, suggesting dissociation between alcohol demand and use behaviors.

**Discussion and Conclusions:** Our results suggest that smokers with AUD were more resistant to price elevations in relation to reducing alcohol consumption as compared to cigarette consumption, suggesting preferential demand for alcohol over cigarettes. However, it is unclear how acute substance exposure/withdrawal impacts the demand indices.

**Scientific Significance:** Potentially differential alcohol and cigarette demands among smokers with AUD should be considered in the concurrent treatment of smoking and alcohol.

## Introduction

Smoking and alcohol misuse often co-occur. In the United States, the prevalence of nicotine dependence among individuals with alcohol dependence is 45.4%, while the prevalence of any alcohol use disorder among adults with nicotine dependence is 22.8% (1). These co-dependent individuals have more difficulty quitting smoking (2). An outstanding problem among those with substance use disorders is their disproportionate valuation of the drug (3) and their disproportionate allocation of resources to obtaining the drug compared to participating in other daily activities (4). This imbalance between drug-related vs. regular activities reflects reinforced drug consumption patterns (5, 6), and the differences in how drugs and nondrug reinforcers (e.g., food) exhibit differential reinforcement strengths can be operationalized using a concept known as Relative Reinforcing Efficacy (RRE).

One validated laboratory approach to measuring the RRE of drugs is hypothetical purchasing tasks, which assess changes in drug purchase and consumption as a function of increasing drug price (7–9). The consumption pattern can yield the demand curve modeled by $Q=Q_{0}*10^{k\left(e^{-\alpha Q_{0}C}-1\right)}$ (10), an exponentiated version of the classic equation by Hursh and Silberberg (11). *Q* represents consumption at price *C*; *Q*_0_ (also referred to as intensity of demand) represents consumption at or near price zero, α represents the rate of change in demand elasticity, and *k* is the span of consumption values in log units. Other demand indices derived from the demand curve include: breakpoint (the price at which consumption reaches 0), O_max_ (the maximum response output or the maximum expenditure), and P_max_ (the price associated with O_max_). P_max_ also indicates the price at which the slope of the demand curve becomes < -1, indicating a shift from relatively inelastic demand where changes in consumption is resistant to increases in price to relatively elastic demand.

Research using the alcohol purchase task (APT) has found alcohol demand to be associated with alcohol use. For example, college students with recent heavy drinking exhibited greater intensity, O_max_, and breakpoint than recent lighter drinkers (9), and the APT's reliability and validity was further confirmed among college students (12). Importantly, heavy drinking smokers exhibited greater O_max_, P_max_, and breakpoint for alcohol compared to heavy drinking nonsmokers (13), suggesting that smoking may increase the demand for alcohol.

Research using the cigarette purchase task (CPT) has suggested that cigarette demand indices are associated with smoking behaviors. Nicotine dependence severity was positively associated with the breakpoint, intensity, P_max_, and O_max_ among young light smokers (8) and among moderately heavy smokers (14). Cigarette demand is also related to psychiatric conditions among smokers. For instance, it was shown that smokers with schizophrenia reported higher intensity, consumption, and expenditure than smokers without schizophrenia (15).

Researchers have further studied the latent structure of the demand indices to identify higher-level factors in the RRE domain that potentially better explain drug use behaviors. Two latent factors, labeled Persistence and Amplitude, have been identified for different drugs, including marijuana (16), alcohol (17), and cigarettes (18–20). The Persistence factor was found to consist of breakpoint, O_max_, P_max_, and elasticity. Higher levels of breakpoint, O_max_, and P_max_, and lower elasticity values were associated with higher Persistence scores, reflecting more persistent demand for the studied drug. However, the Amplitude factor appears to be more heterogeneous. The demand index that loads to this factor is the intensity, and thus it may reflect the maximum possible amount acquired and consumed by users, but other demand indices, such as O_max_ (17, 18) and elasticity (19), were found to load on the Amplitude factor.

While many studies have evaluated the RRE of alcohol and cigarettes separately, most were conducted in nonclinical samples, particularly among younger college students. Smokers with alcohol use disorder (AUD) represent a special population known to be more treatment resistant because of their dual dependency (2). Recently, there have been several attempts studying the demand for alcohol and cigarettes among populations with concurrent use of alcohol and cigarettes. For instance, it was found that smokers showed greater demand for alcohol than nonsmokers among a college student sample (13). Extending these results from university settings to communities, Amlung et al. (21) provided further evidence of increased demand for alcohol among smokers compared to nonsmokers. Recently, in a larger community sample (*n* = 383) of nontreatment seeking heavy drinking smokers, Green et al. (22) found that alcohol and cigarette demand indices were positively correlated and more importantly, they found that compared to alcohol-related dependence measures, smoking-related measures accounted for more variance in alcohol demand's Persistence factor, suggesting that smoking may play a reinforcing role in increasing alcohol demand among nontreatment seeking heavy drinking sample.

These three studies have provided important insights for the interrelationships between the demand for alcohol and cigarettes, shedding light on developing interventions for alcohol and tobacco co-dependence. To complement these findings, we evaluated the demand for alcohol and cigarettes among treatment-seeking smokers with AUD, a clinical population that has not been examined previously. Specifically, the current study used the APT and CPT to examine the baseline demand for alcohol and cigarettes among smokers with AUD enrolled in a clinical trial for the concurrent treatment of AUD and smoking. We aimed to (1) compare the alcohol and cigarette demand indices and their latent factor structures and (2) examine each drug's demand metrics' relationship with the dependence severity of alcohol and nicotine.

## Materials and Methods

### Participants

Participants (*n* = 101) were recruited from the Houston metropolitan area to participate in the current study, as part of a multi-center clinical trial (clinicaltrials.gov # NCT01182766) that used topiramate (23) for the concurrent treatment of both smoking and AUD. Key inclusion criteria included: smoking 5 cigarettes/day or more, producing an expired CO level of ≥ 10 ppm, drinking at least 15 (men) or 8 (women) standard drink units (SDUs) per week, and meeting the DSM-5 (24) diagnosis of mild to severe AUD. Key exclusion criteria included having a medical condition (e.g., seizure disorder) that may put subjects at risk when discontinuing topiramate, or daily use of certain medications (e.g., opioids) that could interact with topiramate. Other exclusions included any psychiatric disorders other than AUD or nicotine dependence, any treatment for alcohol and/or nicotine dependence within 30 days, any illicit drug use, and women who were pregnant or lactating.

The parent study was approved by all participating sites' Institutional Review Boards (IRB), and the current study was approved by the IRB at The University of Texas MD Anderson Cancer Center. All participants provided written informed consent when abstinent from alcohol, as indicated by having a breath alcohol content (BAC) of <0.001%. Participants were not required to abstain from smoking. Instead, they were encouraged to smoke *ab libitum* prior to this session to establish their baseline smoking amount.

### Measures

We used the DSM-IV-based Mini International Neuropsychiatric Interview (MINI, v6.0.0) (25) plus an added question about cravings to homogenize with DSM-5 criteria to determine the diagnosis of AUD or any other psychiatric disorders. The Alcohol Use Disorders Identification Test (AUDIT) (26) assessed alcohol consumption, drinking behaviors, and alcohol-related problems (range: 0–40). The Fagerström Test for Nicotine Dependence (FTND) (27) measured nicotine dependence (range: 0–10). The Short Alcohol Withdrawal Scale (SAWS) (28) assessed the alcohol withdrawal severity (range: 0–30). The Wisconsin Smoking Withdrawal Scale (WSWS) (29) captured smoking withdrawal severity in various subdomains (e.g., concentration and anger). Specifically, we created a consolidated negative affect score to index smoking withdrawal by using the subscales of Anger, Anxiety, Sadness, and Concentration in WSWS. Timeline follow-back interviews recorded alcohol drinking and cigarette smoking amount (30), and the 30-day period preceding the visit date was used to establish the baseline use patterns for the participants. Breath CO and BAC levels were also collected as biochemical indicators of cigarette and alcohol consumption levels.

The purchase tasks were collected at the same session when consent was obtained, before participants were randomized to treatment. The purchase tasks were administered through in-person interview, which allowed our research staff to review the entire instruction with the participants and clarify any outstanding questions. Order of administration was not systematically fixed or counterbalanced. To facilitate comparisons, the instructions of these hypothetical purchase tasks were similar, in which participants were instructed to “imagine a typical day for you that is not in the hospital” and report how drinks or cigarettes they would buy at each price given the following parameters: (1) participant's financial status was the same, (2) there were no other sources of alcohol or cigarettes, (3) any alcohol or cigarettes purchased must be consumed the same day, and (4) alcohol or cigarette craving was the same as they currently felt. The APT defined “a drink” as a standard sized 12-ounce beer, 5-ounce of wine, or 1.5-ounce (shot) of liquor, while the CPT defined cigarettes as individual cigarettes. It should be noted that the APT's instruction was different from previous studies [e.g., (31, 32)], which typically set up the scenario as at a bar or a party during which heavy drinking may be more likely to happen.

Participants then reported the amount of individual drinks or cigarettes at 19 prices: zero, 0.01, 0.02, 0.05, 0.10, 0.25, 0.50, 1, 2, 3, 4, 5, 10, 20, 50, 100, 250, 500, and 1,000 U.S. dollars in an incremental order (8, 33). Due to an oversight, one participant was not administered the purchase tasks.

### Data Processing

We used the “beezdemand” package (34) in the R program (R v3.4.4, The R Foundation for Statistical Computing) to score the purchase tasks.

Non-systematic data were identified using the three-criterion (the trend, bounce, and reversals from zero) algorithm (35). In total, four sessions of APT data and one session of CPT data were identified as non-systematic data and excluded from further analyses. The resulting data were from 99 subjects, consisting of 96 sessions of APT data and 99 sessions of CPT data.

Observed intensity, breakpoint, O_max_, and P_max_ were calculated using the raw data, and these observed values were more reliable than those estimated from the demand curves (12). To compute elasticity, we used the exponentiated version of the model: $Q=Q_{0}*10^{k\left(e^{-\alpha Q_{0}C}-1\right)}$ (10). The *k* values were 3.52 and 2.68 for APT and CPT, respectively, which were computed by subtracting the mean consumption at the lowest price from mean consumption at the highest price with both values log_10_ transformed (19) and then adding 0.5 (34).

For each price, we calculated *Z* scores across all available data with values exceeding 3.29 SD of the mean value (17) considered outliers. In total, 18 outliers (1.85%) were identified. To retain these data, these outliers were recoded as one unit higher than the highest non-outlying value, with the exception of elasticity using 0.1 unit, following previous research (19). By calculating the Mahalanobis distance, one session of CPT data was found to be a multivariate outlier and removed from further analysis. All five demand indices were square-root-transformed to reduce skewness and kurtosis for subsequent data processing and analysis, following previous research (19).

### Statistics

The final data set had data from 99 participants with 96 sessions of APT and 98 sessions of CPT data. All data analyses were conducted using SAS (v9.4; SAS Institute Inc., Cary, NC, USA). To compare the demand indices between APT and CPT, we conducted one-sample paired *t*-tests in SAS with a two-sided alternative. For these tests, a significance level of 0.01 was set to adjust for multiple comparisons involving five separate demand metrics (i.e., the Bonferroni correction). To identify the latent factors for the demand curve indices, we conducted principal component analyses (PCA) with the oblique rotation, which allowed the estimation of multifactorial solutions with correlated factors (17, 18). The scree plot for clear discontinuities between succeeding factors was used for factor retention (eigenvalues ≥ 1.0). A loading of 0.32 was considered to load significantly on a given factor (19).

To examine the correlations between various dependence variables and demand indices, we conducted bivariate correlation analysis. The correlation analysis also included factor scores, which were computed from the five demand indices using the regression method.

## Results

### Sample Characteristics

On average, participants were in their late forties (*M* = 47.0, *SD* = 10.6), mostly male (70%), and moderately dependent on alcohol and nicotine, with a mean score of 19.7 (*SD* = 6.8) and 5.6 (*SD* = 2.2) for the AUDIT and FTND, respectively (see Table 1). At baseline, participants smoked 18.3 (*SD* = 8.4) cigarettes per day and drank 7.9 (*SD* = 4.3) SDUs per day. Table 1 also lists other baseline characteristics, including withdrawal scores and other drinking and smoking behaviors. Overall, our participants represent a heavy drinking and smoking sample.

**Table 1:** Demographics and baseline characteristics (*n* = 99).

**Variable**	**Mean (SD)**
Age in years	47.0 (10.6)
Cigarettes per day	18.3 (8.4)
Cigarettes per smoking day	18.4 (8.3)
CO in ppm	16.9 (9.2)
Hours since last cigarette	1.8 (1.9)
Drinks per day in SDU	7.9 (4.3)
Drinks per drinking day in SDU	10.1 (5.6)
Heavy drinking days	12.9 (11.4)
FTND total score	5.6 (2.2)
AUDIT total score	19.7 (6.8)
WSWS, negative affect	20.8 (12.0)
SAWS	1.22 (0.62)
	***N*** **(%)**
Women	30 (30.3)
Race/ethnicity	
African American	38 (38.4)
European American	51 (51.5)
Hispanic	8 (8.1)
Other	2 (2.0)
Married/cohabitating	30 (30.3)
Employed	63 (63.6)
Alcohol use disorder	
Moderate or severe	90 (90.9)
Mild	9 (9.1)

*CO, Carbon Monoxide; ppm, parts per million; FTND, Fagerström Test for Nicotine Dependence; AUDIT, Alcohol Use Disorders Identification Test. Cigarettes per smoking day, drinks per drinking day, and heavy drinking days (men: >4 daily SDU; women: >3 daily SDU) were calculated over the 30-day period before the visit; WSWS, Wisconsin Smoking Withdrawal Scale; SAWS, Short Alcohol Withdrawal Scale*.

### Alcohol and Cigarettes Demand Indices

The five demand indices for APT and CPT are listed in Table 2. We found that the breakpoint (*t* = 7.89, *p* < 0.0001), O_max_ (*t* = 3.01, *p* < 0.005, and P_max_ (*t* = 6.65, *p* < 0.0001) of the APT were all significantly higher than those of the CPT, while both the elasticity (*t* = −5.39, *p* < 0.0001) and the intensity (*t* = −10.52, *p* < 0.0001) of the APT was lower than that of the CPT.

**Table 2:** Means of alcohol and cigarette demand indices.

**Index**	**APT**	**CPT**
Intensity	8.72 (1.49)	21.29 (1.26)
Elasticity	0.0048 (0.0031)	0.011 (0.0051)
O_max_	18.80 (2.46)	13.80 (4.73)
P_max_	5.99 (1.52)	2.23 (1.29)
Breakpoint	18.10 (5.28)	6.22 (3.19)

*The parentheses list the standard deviations for each index. All the values have been transformed back to their raw values for better interpretability*.

### Latent Structure of the Demand Indices

For the APT, the PCA revealed a two-factor structure, which in total accounted for 80.65% of the variance (Table 3). The first factor explained 52.55% of the variance, and included breakpoint (factor loading: 0.799), O_max_ (0.952), P_max_ (0.693), and elasticity (−0.791), while the second factor explained 28.10% of the variance, and included intensity (0.937), P_max_ (−0.538), and breakpoint (−0.386).

**Table 3:** Latent structures of the alcohol purchase task and cigarette purchase task.

	**APT**		**CPT**	
	**Factor 1**	**Factor 2**	**Factor 1**	**Factor 2**
Intensity	0.218	**0.937**	−0.176	**0.954**
Elasticity	**−0.791**	−0.276	**−0.382**	**−0.658**
O_max_	**0.952**	0.221	**0.727**	**0.342**
P_max_	**0.692**	**−0.538**	**0.983**	−0.111
Breakpoint	**0.799**	**−0.386**	**0.955**	−0.070

*Factor loadings are shown in this table and those >0.32 are highlighted in bold. For both tasks, square-root-transformed values were used for the Principal Component Analysis. Each task yielded a two-factor solution*.

For the CPT, the PCA revealed a two-factor structure, which in total accounted for 73.32% of the variance (Table 3), but had differential loadings from the demand indices of the APT. The two factors explained 46.67 and 26.65% of the variance. The first factor was mainly loaded by breakpoint (0.955), O_max_ (0.727), P_max_ (0.983), and elasticity (−0.382), while the second factor was mainly loaded by O_max_ (0.342), elasticity (−0.658), and intensity (0.953).

Despite differential loadings of the demand indices to their respective factors, these two-factor solutions for both tasks have qualitative similarities. Specifically, the first factor had significant loadings from breakpoint, O_max_, P_max_, and elasticity, which reflect the persistent drug use pattern, and thus we referred to Factor 1 as the Persistence factor. The second factor had significant loadings from intensity, which reflects the drug consumption levels, and thus we termed Factor 2 the Amplitude factor.

### Correlation Analysis

As shown in Table 4, baseline drinking levels were significantly correlated with AUDIT scores and BAC levels (both Pearson's *r* > 0.35, *p* < 0.01). Cigarette-related dependence measures (i.e., FTND scores, baseline smoking levels, CO) were also correlated with each other (*p* < 0.05). However, the alcohol withdrawal measure SAWS scores were not correlated with any other alcohol-related dependence measures, and the smoking withdrawal measure WSWS negative affect scores were not correlated with any other cigarette-related dependence measures. We did not find any correlations between alcohol- and cigarette-related dependence measures with the exception that WSWS negative affect scores were correlated with AUDIT and SAWS scores.

**Table 4:** Bivariate correlations between demand metrics and dependence measures.

**Measure**	**Dependence Measures**								**Demand Metrics**													
	**Alcohol**				**Cigarettes**				**APT**							**CPT**						
	**(1)**	**(2)**	**(3)**	**(4)**	**(5)**	**(6)**	**(7)**	**(8)**	**(9)**	**(10)**	**(11)**	**(12)**	**(13)**	**(14)**	**(15)**	**(16)**	**(17)**	**(18)**	**(19)**	**(20)**	**(21)**	**(22)**
(1). AUDIT	–	0.36^**^	0.09	0.17	0.05	0.06	0.02	0.27^**^	0.07	0.09	0.03	−0.04	0.05	0.08	0.02	−0.04	−0.02	−0.01	0.02	0.001	−0.03	−0.01
(2). Baseline Drinking			0.39^**^	0.02	0.06	0.17	−0.18	0.02	−0.05	0.05	−0.02	−0.05	0.11	0.02	0.11	−0.04	−0.03	−0.10	−0.05	0.03	−0.06	0.05
(3). BAC				0.08	0.05	0.06	0.05	0.07	0.007	0.11	0.04	−0.06	−0.05	0.06	−0.02	−0.07	−0.03	−0.10	−0.02	0.10	−0.09	0.08
(4). SAWS					−0.004	−0.08	−0.08	0.41^**^	0.29^**^	0.32^**^	0.18	−0.16	0.08	0.30^**^	−0.001	0.19	0.15	0.15	−0.02	0.01	0.17	0.01
(5). FTND						0.59^**^	0.20^*^	0.13	0.20^*^	0.07	0.18	−0.14	−0.04	0.16	−0.10	0.24^*^	0.42^**^	0.20^*^	−0.35^**^	0.49^**^	0.28^**^	0.51^**^
(6). Baseline Smoking							0.30^**^	0.11	0.26^*^	0.13	0.28^**^	−0.23^*^	−0.15	0.25^*^	−0.20	0.14	0.34^**^	0.07	−0.44^**^	0.55^**^	0.17	0.60^**^
(7). CO								−0.04	0.19	0.06	0.12	−0.14	−0.14	0.13	−0.15	0.15	0.16	0.07	−0.19	0.12	0.13	0.17
(8). WSWS									0.34^**^	0.28^**^	0.38^**^	−0.09	−0.11	0.31^**^	−0.23^*^	0.19	0.26^*^	0.22^*^	−0.20^*^	0.16	0.24^*^	0.20^*^
(9). APT Breakpoint										0.70^**^	0.87^**^	−0.42^**^	−0.22^*^	0.86^**^	−0.52^*^	0.44^**^	0.39^**^	0.41^**^	−0.32^**^	0.22^*^	0.44^**^	0.27^**^
(10). APT O_max_											0.58^**^	−0.64^**^	0.25^*^	0.92^**^	0.07	0.32^**^	0.33^**^	0.31^**^	−0.37^**^	0.18	0.35^**^	0.28^**^
(11). APT P_max_												−0.35^**^	−0.35^**^	0.78^**^	−0.65^**^	0.37^**^	0.38^**^	0.33^**^	−0.34^**^	0.19	0.39^**^	0.27^**^
(12). APT Elasticity													−0.12	−0.75^**^	−0.15	−0.19	−0.23^*^	−0.14	0.47^**^	−0.18	−0.23^*^	−0.33^**^
(13). APT Intensity														0.07	0.90^**^	0.09	−0.002	0.08	−0.08	0.13	0.06	0.10
(14). APT Persistence^a^															−0.16	0.40^**^	0.40^**^	0.36^**^	−0.45^**^	0.24^*^	0.42^**^	0.36^**^
(15). APT Amplitude^b^																−0.09	−0.14	−0.09	−0.004	0.03	−0.11	0.01
(16). CPT Breakpoint																	0.65^**^	0.88^**^	−0.46^**^	0.13	0.93^**^	0.23^*^
(17). CPT O_max_																		0.71^**^	−0.66^**^	0.33^**^	0.83^**^	0.57^**^
(18). CPT P_max_																			−0.42^**^	0.09	0.95^**^	0.20
(19). CPT Elasticity																				−0.45^**^	−0.59^**^	−0.78^**^
(20). CPT Intensity																					0.12	0.90^**^
(21). CPT Persistence^a^																						0.31^**^
(22). CPT Amplitude^b^																						–

*AUDIT, Alcohol Use Disorder Identification Test; BAC, Breadth Alcohol Concentration; SAWS, Short Alcohol Withdrawal Scale; FTND, Fagerstrom Test for Nicotine Dependence; CO, Carbon Monoxide in ppm; WSWS, Wisconsin Smoking Withdrawal Scale, Negative Affect Score; APT, Alcohol Purchase Task; CPT, Cigarette Purchase Task*;

a *Persistence Factor Score*;

b *Amplitude Factor Score*.

* *p < 0.05*;

** *p < 0.01*.

Most of the bivariate correlations between alcohol and cigarette demand metrics were significant. However, alcohol demand metrics were not correlated with any alcohol-related dependence measures except for the SAWS scores (Pearson's *r* > 0.25, *p* < 0.01). In light of these null findings, we also examined alcohol demand metrics in relationship with the MINI-based (25) categorical alcohol diagnosis measure, but found that alcohol diagnostic category had no relationship with alcohol demand indices.

By contrast, most of the cigarette demand metrics were significantly correlated with smoking-related dependence measures, particularly FTND scores (Pearson's *r* > 0.20, *p* < 0.05), baseline smoking levels (Pearson's *r* > 0.21, *p* < 0.05), and WSWS negative affect scores (Pearson's *r* > 0.20, *p* < 0.05).

## Discussion

Our finding that participants had higher O_max_ (expenditure) and elasticity (insensitivity to price increase) in the APT than in the CPT suggests that they were willing to allocate more economic resources toward alcohol than cigarettes and were less sensitive to the price escalation of the alcohol than that of cigarettes. These results suggest that alcohol had relatively greater RRE than cigarettes among smokers with alcohol use disorder. Our results were consistent with an earlier study among alcohol-dependent individuals (36). They used a multiple-choice questionnaire to assess the crossover point between drug (alcohol or cigarettes) and monetary values and found that the crossover point for the monetary option was higher for a drink than for a cigarette, suggesting that alcohol had greater RRE than cigarettes did among a similar population.

The greater values of O_max_ and lower elasticity scores in the APT than those in the CPT suggested that smokers with AUD had greater demand for alcohol than cigarettes. Consistent with difference in elasticity between alcohol and cigarette demand, our findings support the notion that smokers with AUD were more resistant to the price elevation in terms of reducing their alcohol consumption compared with their cigarette consumption. Notably, greater and more sustained demand for alcohol may be related to one's smoking status *per se*, as previous research showed that heavy drinking smokers reported greater alcohol demand than heavy drinking nonsmokers (13, 21). Although our participants reported lower intensity of alcohol than that of cigarettes, this difference in intensity may reflect the inherent difference in characteristics between alcohol and cigarettes, such as packaging and consumption patterns specific to the products. The relative difference in intensity between alcohol and cigarettes demand, as well as their relative difference in baseline consumption patterns (i.e., less alcohol consumption measured in daily SDU than cigarette consumption measured in cigarettes per day) is consistent with previous research using a similar sample—heavy drinking smokers (22).

Our PCA suggested a robust two-factor latent structure for the APT that accounted for 80.65% of the variance. This finding is consistent with previous research that identified a two-factor solution for marijuana (16), alcohol (17), and cigarettes (18–20). Moreover, consistent with these studies, the first factor includes breakpoint, O_max_, P_max_, and elasticity for both alcohol and cigarette demands. These four indices reflect the sensitivity to the increasing prices of alcohol and cigarettes. Thus, this factor indicates the persistence of alcohol and cigarette use behaviors among this population.

The second factor has been commonly referred to as Amplitude (16–18, 20), which reflects individuals' consumption levels when the cost was minimum. This factor was mainly attributable to the intensity index. However, previous research identified differential contributions from a second demand index. Three studies found extra loading from O_max_ (17, 18, 20), one study found elasticity (19), and one found no extra indices (16). Unlike these studies, we found that the Amplitude factor had extra loading from the breakpoint and P_max_, although three studies found similar nonsignificant negative loadings from P_max_ (16, 19, 20). These results highlight the heterogeneity of the second factor, despite the consistent loading from intensity.

For the cigarette demand's PCA, we replicated a two-factor (18–20). Overall, the loadings to the first factor were similar to our findings with the APT's PCA. However, the Persistence factor accounted for 52.55% of the variance in alcohol demand vs. 46.67% of the variance in cigarette demand, which suggests that smokers with AUD are characterized by higher persistence use of alcohol than cigarettes, consistent with the differences of O_max_ and elasticity between APT and CPT.

Perhaps the most interesting finding with the cigarette demand's PCA was the second factor. This factor pattern is unique because it has been partially reported. For example, Bidwell et al. (18) and O'Connor et al. (20) reported O_max_, while González-Roz et al. (19) reported elasticity to load to the second factor. Except for the same factor (i.e., intensity) loading to the second factor, the loading from the other four demand indices have a complementary pattern (breakpoint and P_max_ for APT vs. O_max_ and elasticity for CPT). These differential loading patterns highlight the heterogeneity of the Amplitude factor, and distinct latent factors may contribute to the observed differential demand for alcohol and cigarettes.

We found that cigarette demand indices were significantly correlated with FTND scores, baseline smoking rate, and smoking withdrawal (i.e., WSWS negative affect scores). These positive correlations have been reported in several studies (8, 14, 15, 37, 38), and suggest that smokers who were more dependent on nicotine have more demand for cigarettes. Notably, the correlations between cigarette demand indices (i.e., O_max_, P_max_, elasticity) and WSWS negative affect scores have not been previously reported. Although our participants were relatively satiated with smoking when they completed the CPT, these findings suggest that smokers' withdrawal experience was positively associated with their demand for cigarettes.

In contrast, we did not find alcohol demand indices and latent factors were correlated with alcohol-dependence measures except for the SAWS scores. Several studies have reported positive correlations, such as drinks per week (21, 38, 39), monthly binge drinking days (39), and AUDIT scores (38, 40). Although the exact reasons for this discrepancy are unclear, we speculate that two factors may be relevant. The first is that the APT used in our study is different from other studies in terms of its instruction about framing the hypothetical drinking context, which will be discussed more in the study limitations later. Briefly, our generic description of the drinking situations may be insufficient to allow participants to imagine their typical drinking scenarios (e.g., bar) thus that they could not accurately report their alcohol demand. The other possible reason might be differences between study populations. Unlike previous studies (21, 22), our participants were treatment seeking, and thus their motivation of quitting/reducing drinking and smoking may have changed how they responded in these purchase tasks. Besides the difference of motivations, our participants were more dependent on alcohol than the undergraduate samples tested previously (40)—the average AUDIT score in our sample was almost twice that of theirs. Similarly, all of our participants had a diagnosis of AUD, while only about 50% who were dependent or abusing alcohol in the study by Amlung et al. (21). Additionally, our participants were also heavy smokers and importantly, several studies found that smoking resulted in higher demand for alcohol than nonsmoking (13, 21, 22). Thus, smoking may have resulted in a higher and more uniform alcohol use demand, masking a possible linear relationship between dependence and demand. Consistent with this possibility, we did not find any relationships between alcohol demand and alcohol misuse diagnoses. Although this possibility exists, future studies evaluating this population (i.e., treatment-seeking smokers with AUD) will help address whether heavy smoking can indeed mask the relationship between alcohol dependence measures and alcohol demand indices.

The positive correlations between alcohol and cigarette demand indices suggest that those who had higher demand for alcohol tended to have higher demand for cigarettes too. This co-demand pattern is consistent with a recent study (22) which revealed the same positive correlations among a similar sample of heavy drinking smokers (but who were not seeking treatment). Moreover, by conducting hierarchical multiple regression analyses, their study found that smoking had a positive impact on the alcohol demand, but not the other way around (22). Their finding may help explain the relative higher demand for alcohol than for cigarettes among treatment-seeking smokers with AUD in the present study, because our participants were more dependent on nicotine (FTND = 5.6, CPD = 18.3) than those nontreatment seeking heavy drinking smokers (FTND = 4.4, CPD = 14.0) in their study (22)—the relatively higher level of smoking in our sample may have resulted in greater alcohol demand in an asymmetric fashion.

An important study factor that should be taken into account is the differential alcohol and smoking satiation statuses among the participants. Although our participants were instructed to complete the hypothetical purchase tasks in a general context, we cannot rule out the possibility that the reported demand patterns may have been influenced by their alcohol and smoking statuses. Previously, we speculated that the special characteristics (e.g., heavy alcohol use) may have caused the null correlations between alcohol demand and alcohol-related measures. Unlike other alcohol-related measures, alcohol withdrawal scores were correlated with alcohol demand metrics, which support the possibility that alcohol deprivation status may have indeed increased the reported demand for alcohol among our participants who experienced more alcohol withdrawal, consistent with a previous study which showed the increased cigarette demand among nicotine-deprived smokers (41). In the current study, we also found that cigarette demand metrics were positively correlated with smoking withdrawal, which suggests an increased demand for cigarettes due to smoking deprivation. However, the exact effects of alcohol deprivation on alcohol demand are more speculative with the current study design (e.g., all participants were deprived of alcohol), which can be examined in future studies that contrast the alcohol demand metrics between deprived and satiated patients with AUD.

The study has the following limitations. First, the APT and CPT were administered separately, with each having no assumption of allocating limited resources to the other. Although our findings suggested that alcohol had higher demand than cigarettes using the single-commodity tasks (i.e., APT and CPT), we do not have direct evidence that alcohol is preferred if both drugs are considered in the same context. Such relative preference between two co-used drugs can be best captured by a cross-commodity task wherein the consumption patterns for both drugs are examined simultaneously. Using the cross-commodity paradigm, researchers have found a complex interplay between cannabis and alcohol use with nontrivial proportions of the study sample (i.e., adult past-month cannabis and alcohol users) showing patterns of complementarity, substitution, and independence (42). However, in a different cross-commodity study involving marijuana and tobacco cigarettes, researchers found an independent demand pattern between these two drugs (43). These studies suggest the manipulation robustness of using the cross-commodity paradigm in substance use research to simultaneously study co-use of drugs. More importantly, this paradigm provides a better ecological validity by placing participants in a more realistic context with their access to both drugs while having limited shared resources. Future studies should consider using this cross-commodity paradigm to better capture the demand for alcohol and cigarettes among smokers with AUD, which may shed light on developing personalized treatments based on relative demand patterns between alcohol and cigarettes.

Second, to make the participants have similar contexts for the APT and CPT, the APT's instruction used the same contextual description as the CPT's, and differences in the current APT's instructions from previous studies (31) may have affected participants' ability to report their alcohol demand with ecological validity. Previous studies have generally assessed alcohol demand under contexts in which alcohol is likely to be consumed (e.g., at a bar during peak drinking times). Similarly, time parameters such as duration of access (31) and weekend vs. weekday (44) have been shown to impact alcohol demand.

Third, per protocol requirements, participants were abstinent from alcohol to have proper cognitive functionality to complete the visits, but they could smoke *ad libitum*. Thus, differences in alcohol deprivation and smoking satiation may have affected the demand for alcohol and cigarettes.

Alcohol appeared to have higher relative reinforcing efficacy than cigarettes among adult smokers with alcohol use disorder, as evidenced by their greater demand for alcohol than for cigarettes, although it is possible that acute substance status may play a role in modulating the demand for alcohol and cigarettes. A two-factor structure was identified for both alcohol and cigarette demand curves, and the differential loadings of demand indices in the current population of heavy drinking smokers and other less dependent younger samples assessed previously suggest a distinct demand pattern for smokers with AUD. As an important future direction of the present study, hierarchical multiple regressions analyses of multiple purchase tasks (21, 22) should be conducted to provide a deeper understanding of cross-substance demand for alcohol and cigarettes among treatment-seeking smokers with AUD.

## Data Availability Statement

The raw data supporting the conclusions of this article will be made available by the authors, without undue reservation.

## Ethics Statement

The studies involving human participants were reviewed and approved by University of Texas MD Anderson Cancer Center Institutional Review Board. The patients/participants provided their written informed consent to participate in this study.

## Author Contributions

JR designed the current study and wrote the protocol. NA-D, RA, and PC designed the parent clinical study and developed its protocol. MK-H provided medical oversight for the entire study. JY provided expertise on implementing, analyzing, and interpreting the behavioral economics tasks. YC, PL, and JR wrote the first draft of the manuscript. All other authors edited the manuscript and approved the final manuscript.

## Conflict of Interest

The authors declare that the research was conducted in the absence of any commercial or financial relationships that could be construed as a potential conflict of interest.
